# Validation of self‐reported medication use for hypertension, diabetes, and dyslipidemia among employees of large‐sized companies in Japan

**DOI:** 10.1002/1348-9585.12138

**Published:** 2020-07-25

**Authors:** Kota Fukai, Tomohisa Nagata, Koji Mori, Makoto Ohtani, Kenji Fujimoto, Masako Nagata, Yoshihisa Fujino

**Affiliations:** ^1^ Department of Preventive Medicine Tokai University School of Medicine Isehara City Japan; ^2^ Department of Occupational Health Practice and Management Institute of Industrial Ecological Sciences University of Occupational and Environmental Health Kitakyushu Japan; ^3^ Data Science Center for Occupational Health University of Occupational and Environmental Health Kitakyushu Japan; ^4^ Department of Public Health University of Occupational and Environmental Health Kitakyushu Japan; ^5^ Department of Environmental Epidemiology Institute of Industrial Ecological Sciences University of Occupational and Environmental Health Kitakyushu Japan

**Keywords:** health checkup, occupational health, self‐administered questionnaire, validity

## Abstract

**Objective:**

The aim of this study was to evaluate the validity of self‐reported medication use for hypertension, diabetes, and dyslipidemia by comparison with health insurance claims among employees of large‐sized companies in Japan.

**Methods:**

Participants were 61 676 participants of 13 large‐sized companies in Japan. Self‐reports on medication use were obtained through web‐ or paper‐based questionnaires conducted at the annual health checkup in fiscal year 2016. Health insurance claims for medication were obtained from corporate health insurance associations from April 1, 2016, to March 31, 2017. Agreement rate, sensitivity, specificity, positive and negative predictive values (PPV and NPV), and kappa statistics of self‐reporting were examined for different reference periods (1‐, 2‐, and 3‐ months, and 1‐year). Subgroup analysis was conducted stratified by sex, age, body mass index, smoking, alcohol drinking, blood pressure, hemoglobin A1c, and low‐density lipoprotein cholesterol.

**Results:**

Agreement, sensitivity, specificity, PPV, and NPV were 0.98, 0.90, 0.98, 0.87, and 0.99 for hypertension, 0.99, 0.89, 1.00, 0.89, and 1.00 for diabetes, and 0.98, 0.86, 0.99, 0.83, and 0.99 for dyslipidemia, respectively, between self‐reports and claims data for 3 months. Kappa statistics were highest with the 3‐month reference period of claims data for hypertension, diabetes, and dyslipidemia. No major concordance was observed between the subgroups.

**Conclusion:**

This validation of self‐reported medication use for hypertension, diabetes, and dyslipidemia showed almost perfect reliability among employees of large‐sized companies in Japan.

## 1 INTRODUCTION

Evaluation of current medication use by individuals for common diseases such as hypertension, diabetes, and dyslipidemia provide essential information for occupational health professionals in carrying out health management in companies. Information on whether an individual uses a medication often affects occupational health professionals if the employee is eligible for further interventions, such as health guidance.^1^ Moreover, failure to properly evaluate medication use for common diseases among employees as a whole can affect future health promotion plans and measures in companies.^2^ Medication use is often treated as an exposure or outcome factor, as well as a confounding factor, in epidemiological studies.^3^ It is therefore important to evaluate current medication use precisely.

Medication use is generally assessed using self‐administered questionnaires,^4^, ^5^, ^6^, ^7^ These allow the collection of respondent health information, including medication use, general health status, lifestyle factors, and medical history, at a single time in situations such as health checkups taken by large numbers of people.^8^ However, self‐report data has been shown to be affected by measurement error, such as due to recall bias, misinterpretation of questions, and reporting bias.^9^

In contrast, healthcare insurance claims represent objective data that are considered the “gold standard” in identifying medication use.^3^ Although the validity of self‐reports on medication use has been evaluated against national or regional government healthcare claims data in various countries,^1^, ^10^, ^11^, ^12^ results are inconsistent. A recent systematic review reported that sensitivity for medications for common chronic diseases ranged from 48% to 93% against a method that refers to a pharmacy database for a certain period of time.^3^ In Japan, only one large‐scale validation study of self‐reported medication has appeared, and this was conducted in a population of local residents.^1^ No study has been conducted in a workplace setting. Nevertheless, validity can vary not only by country or region but also by residential or occupational setting.

In this study, we examined the agreement between medication use self‐reported during annual health checkups at large‐sized Japanese companies with prescribing data from health insurance claims as an objective standard for commonly used medications for three conditions, hypertension, diabetes, and dyslipidemia. We also conducted sub‐group analyses to examine whether agreement differed by participant characteristics. To our knowledge, this is one of the largest epidemiological studies of to examine the validation of self‐reported medication use, and the first in an occupational setting in Japan.

## 2 METHODS

### 2.1 Study design and participants

We used the data from participants of the “the Collabo‐Health Study Group,” established in April 2014, an organization composed of 13 pharmaceutical or manufacturing companies, most of which are listed on the Tokyo Stock Exchange First Section, and their related health insurance unions. Details of this study are reported elsewere.^13^ Briefly, the Collabo‐Health Study Group collects health checkup data and health insurance claims from all participating employees annually. During each health checkup, the participants answer web‐ or paper‐based questionnaires in several domains, including medication use, medical history, personal health status, and lifestyle factors. Employees were free to choose whether to participate. The study design was explained to employees and employers via email, intranet homepage, or the committee of occupational health and safety in each company and health insurance union. The study was approved by the ethics committee of the University of Occupational and Environmental Health, Kitakyushu, Japan (Protocol Number H26‐026).

For this study, we obtained data of medication use self‐reports for hypertension, diabetes, or dyslipidemia from May 1, 2016 to March 31, 2017 and pharmacy claims data from the corporate health insurance associations from April 1, 2016 to March 31, 2017. Although we obtained self‐reports from subjects whose health checkup was in April 2016, we did not include them in this study due to differences in validity between the different reference periods for claims data, as described below. For this study, 65 306 participants (50 265 men and 15 041 women) were subject to analysis. Among the study subjects, we excluded those with incomplete data for self‐reports (n = 3630). Although the participation rate of the annual health checkups is considered to be almost 100% in such companies, the questionnaire used a government‐form, which is indicated for patients over 40 years old. Sixty‐two percent of the subjects excluded were under 40 years old. Finally, a total of 61 676 participants (47 362 men and 14 314 women) remained for analysis. The mean (standard deviation; minimum‐maximum) number of the participants of the companies was 4744 (2748; 2174‐12 519).

### 2.2 Self‐administered questionnaire

The self‐reports by questionnaire were filled in by employees at the time of the annual health checkup at each workplace. Participants were asked about their current medication use for treatment of hypertension, diabetes, or dyslipidemia (“Are you currently taking medications to lower blood pressure?”; “Are you currently taking medication to lower blood sugar, either insulin injection and/or oral medication?”, “Are you currently taking medication for hypercholesterolemia?”), and answered “yes” or “no” to each question. If the participant answered “yes” to either of the questions, the self‐report was determined to be positive.

### 2.3 Health insurance claims for medication

We received pharmacy claims data on medication use from the corporate health insurance associations of all participants from April 1, 2016, to March 31, 2017. Health insurance claims data had an individual code which allows merging with the health checkup data. Medications appropriate for hypertension, diabetes, and dyslipidemia were identified using the code of the National Health Insurance Drug List (NHI code), which is managed by the Ministry of Health, Labour and Welfare, Japan.^14^ This is a 12‐digit alphanumeric code assigned to each drug; the first four digits define the medicinal effect and next three define the administration route (eg, oral or injection). For hypertension, we identified those who had prescriptions for orally administered medications with an NHI code beginning with “213” (diuretics), “214” (antihypertensives), “2123” (beta‐blockers), “217” (vasodilators), and “2190” (other circulatory agents); for diabetes, we identified those who had prescriptions for orally administered medications with a code beginning with “396” (diabetes agents) and injected medications beginning with “2492” (pancreatic hormone preparation); and for dyslipidemia we identified those who had prescriptions for orally administered medications with a code beginning with “218” (hypercholesterolemia agents). Health insurance claims data are tabulated by month, and if a prescription for more than one day was identified in that month, the prescription was determined to be positive for that month.

### 2.4 Other variables

Additional information collected in the questionnaire during the health checkup included sex (men or women), age (years), smoking (current smoker or not), and alcohol drinking (heavy drinker [consuming more than 46 g of ethanol per day] or not). Body mass index (BMI, kg/m^2^) was calculated from anthropometric measurement of body weight and height by medical staff. Systolic and diastolic blood pressure (mmHg) were measured by medical staff according to the standard protocol of each health checkup organizations, and if more than one measurement was taken, the lowest blood pressure value was adopted. Hemoglobin A1c (%), and low‐density lipoprotein (LDL) cholesterol was measured using fasting blood samples collected during the health checkup.

### 2.5 Statistical analysis

We defined three different reference periods for claims data on medication use to allow for the different lengths of prescription by physicians: one month, namely the month of the health checkup (“1 month”); past two months, including the month of the health checkup (“2 months”); and past three months, including the month of the health checkup (“3 months”). We also defined the annual fiscal year in which the health checkup occurred (“1 year”). The validity of the self‐reports from questionnaires was assessed by comparison with prescription data from pharmacy insurance claims using the agreement rate (1 − (false‐positive rate) − (false‐negative rate)), sensitivity, specificity, and positive and negative predictive values (PPV and NPV).^15^ In addition, we also calculated kappa statistics for each reference period. The kappa statistic is scaled to 0 when agreement is expected by chance and to 1 when agreement is perfect. Interpretation of kappa was based on Landis and Koch's classifications, namely 0.0‐0.2 as slight, 0.21‐0.40 as fair, 0.41‐0.60 as moderate, 0.61‐0.80 as substantial, and 0.81‐1.00 as almost perfect.^16^

We also conducted subgroup analyses stratified by sex (men or women), age (<40, 40 to 49, or ≥50 years), BMI (<18.5, 18.5 to <25.0, or ≥25.0 kg/m^2^), smoking (current smoker or not), alcohol drinking (heavy drinker or not), blood pressure (systolic < 120 and diastolic <80, systolic <140 and/or diastolic <90, systolic <160 and/or diastolic <100, or systolic ≥160 or diastolic ≥100 mmHg), hemoglobin A1c (<6.5%, 6.5% to <8.0%, or ≥8.0%), and LDL cholesterol (<120, 120 to <140, 140 to <160, and ≥160 mg/dL). All analyses were performed using Statistical Analysis System Software Version 9.4 (SAS Institute, Cary, NC, USA).

## 3 RESULTS

The 61 676 participants in the study are characterized in Table 1 by sex. Three‐quarters of the participants were men. Current smoking rate was 31.8% in men and 10.2% in women. Among the participants, 13.9% of men and 5.2% of women had hypertension (systolic ≥140 or diastolic ≥90 mmHg), 3.9% of men and 1.1% of women had suspected diabetes (hemoglobin A1c ≥6.5%), and 26.9% of men and 17.3% of women had hyper‐LDL cholesterolemia (LDL cholesterol ≥140 mg/dL).

**Table 1:** Participant characteristics

	Men		Women	
	N	(%)	N	(%)
All (N = 61 676)	47 362	(76.8)	14 314	(23.2)
Age, y				
<40	10 243	(21.6)	5547	(38.8)
40 to 49	18 342	(38.7)	5556	(38.8)
≥50	18 777	(39.7)	3211	(22.4)
BMI, kg/m^2^				
<18.5	1351	(2.9)	2355	(16.5)
18.5 to <25.0	32 286	(68.2)	10 035	(70.1)
≥25.0	13 725	(29.0)	1924	(13.4)
Current smoker	15 036	(31.8)	1463	(10.2)
Heavy drinker^a^	4726	(10.0)	317	(2.2)
Blood pressure^b^, mmHg				
Systolic < 120 and diastolic < 80	21 095	(44.5)	10 585	(74.0)
Systolic < 140 and/or diastolic < 90	19 729	(41.7)	2992	(20.9)
Systolic < 160 and/or diastolic < 100	5093	(10.8)	568	(4.0)
Systolic ≥ 160 or diastolic ≥ 100	1444	(3.1)	166	(1.2)
Hemoglobin A1c^b^, %				
<6.5	42 139	(89.0)	12 800	(89.4)
6.5 to <8.0	1409	(3.0)	118	(0.8)
≥8.0	415	(0.9)	46	(0.3)
LDL cholesterol^b^, mg/dL				
<120	22 107	(46.7)	8396	(58.7)
120 to <140	11 427	(24.1)	2601	(18.2)
140 to <160	7531	(15.9)	1479	(10.3)
≥160	5195	(11.0)	1004	(7.0)

Abbreviation: BMI, body mass index; LDL, low‐density lipoprotein.

^a^ Heavy drinker was defined as consumption of more than 46 g of ethanol per day.

^b^ Missing data existed for blood pressure (n = 4), hemoglobin A1c (n = 4749), and LDL cholesterol (n = 1936).

Table 2 shows the number of participants by self‐report and claims data prescription status for medication use by each reference period. For hypertension, 4.4% of the total population self‐reported positive use of medication but had no prescription in the health insurance data during the 1‐month reference period. The same status was observed in 1.0% for diabetes, and 3.4% for dyslipidemia. These rates decreased when the reference period was lengthened. Table 3 shows the indicators for validity of the self‐report by reference period. Sensitivity and specificity were all >80% for hypertension, diabetes, and dyslipidemia, except for the 1‐year reference period. PPV was around 60% for the 1‐month reference period for hypertension, diabetes, and dyslipidemia, but close to 90% for the 3‐month period. The kappa values were substantial for the 1‐month reference period, almost perfect for 2 months or more, and highest for 3 months. We therefore used the results for the 3‐month reference period for further subgroup analyses.

**Table 2:** Number of participants by status of self‐report and prescriptions from claims data for medication use by reference period

	All	Self‐report (+)^f^				Self‐report (−)^f^			
		Prescription (+)^f^		Prescription (−)^f^		Prescription (−)^f^		Prescription (+)^f^	
	N	N	(%)	N	(%)	N	(%)	N	(%)
Hypertension^a^									
1 mo	61 676	3888	(6.3%)	2691	(4.4%)	54 684	(88.7%)	413	(0.7%)
2 mo	61 676	5399	(8.8%)	1180	(1.9%)	54 530	(88.4%)	567	(0.9%)
3 mo^d^	61 676	5714	(9.3%)	865	(1.4%)	54 454	(88.3%)	643	(1.0%)
1 y^e^	61 676	6207	(10.1%)	372	(0.6%)	53 283	(86.4%)	1814	(2.9%)
Diabetes^b^									
1 mo	61 676	1086	(1.8%)	608	(1.0%)	59 861	(97.1%)	121	(0.2%)
2 mo	61 676	1455	(2.4%)	239	(0.4%)	59 815	(97.0%)	167	(0.3%)
3 mo^d^	61 676	1515	(2.5%)	179	(0.3%)	59 800	(97.0%)	182	(0.3%)
1 y^e^	61 676	1628	(2.6%)	66	(0.1%)	59 539	(96.5%)	443	(0.7%)
Dyslipidemia^c^									
1 mo	61 676	2549	(4.1%)	2068	(3.4%)	56 647	(91.9%)	412	(0.7%)
2 mo	61 676	3608	(5.9%)	1009	(1.6%)	56 480	(91.6%)	579	(0.9%)
3 mo^d^	61 676	3850	(6.2%)	767	(1.2%)	56 418	(91.5%)	641	(1.0%)
1 y^e^	61 676	4206	(6.8%)	411	(0.7%)	55 523	(90.0%)	1536	(2.5%)

^a^ Prescriptions of antihypertensive drugs.

^b^ Prescriptions of oral hypoglycemic drugs and/or self‐injected insulin.

^c^ Prescriptions of hypercholesterolemia drugs.

^d^ Participants in May 2016 limited to 2 mo for prescription data due to unavailability of March 2016 health insurance data.

^e^ Prescriptions from April 2016 to March 2017.

^f^ Self‐report (+) or Self‐report (−) indicate that the participant did or did not report medication use; Prescription (+) or Prescription (−) indicate that the claim did or did not exist in the health insurance data during the respective period.

**Table 3:** Validity of self‐reported use of medication by reference period

	Agreement	Sensitivity	Specificity	PPV	NPV	Kappa
Hypertension^a^						
1 mo	0.95	0.90	0.95	0.59	0.99	0.69
2 mo	0.97	0.90	0.98	0.82	0.99	0.85
3 mo^d^	0.98	0.90	0.98	0.87	0.99	0.87
1 y^e^	0.96	0.77	0.99	0.94	0.97	0.83
Diabetes^b^						
1 mo	0.99	0.90	0.99	0.64	1.00	0.74
2 mo	0.99	0.90	1.00	0.86	1.00	0.87
3 mo^d^	0.99	0.89	1.00	0.89	1.00	0.89
1 y^e^	0.99	0.79	1.00	0.96	0.99	0.86
Dyslipidemia^c^						
1 mo	0.96	0.86	0.96	0.55	0.99	0.65
2 mo	0.97	0.86	0.98	0.78	0.99	0.81
3 mo^d^	0.98	0.86	0.99	0.83	0.99	0.83
1 y^e^	0.97	0.73	0.99	0.91	0.97	0.80

Abbreviations: NPV, Negative Predictive Value; PPV, positive predictive value.

^a^ Prescription of antihypertensive drugs.

^b^ Prescription of oral hypoglycemic drugs and/or self‐injected insulin.

^c^ Prescriptions of hypercholesterolemia drugs.

^d^ Participants in May 2016 limited to 2 mo for prescription data due to unavailability of March 2016 health insurance data.

^e^ Prescriptions from April 2016 to March 2017.

Tables 4, 5, 6 show the analyses of subgroups divided by sex, age, BMI, smoking, heavy alcohol drinking, blood pressure, and hemoglobin A1c. In almost all subgroups, sensitivity and specificity were >80% and agreement were >90%, except for age <40 years, BMI <18.5 kg/m^2^, hemoglobin A1c ≥8.0%, and LDL cholesterol ≥160 mg/dL. PPV and NPV were >80% in all subgroups, except for LDL cholesterol ≥140 mg/dL.

**Table 4:** Validity of self‐reported use of medication for hypertension in predicting actual prescription over 3 mo among subgroups

	Hypertension^a^				
	Agreement	Sensitivity	Specificity	PPV	NPV
All	0.98	0.90	0.98	0.87	0.99
Sex					
Men	0.97	0.91	0.98	0.87	0.99
Women	0.99	0.80	1.00	0.89	0.99
Age, y					
<40	0.99	0.61	1.00	0.80	1.00
40 to 49	0.98	0.87	0.99	0.87	0.99
≥50	0.96	0.92	0.96	0.87	0.98
BMI, kg/m^2^					
<18.5	0.99	0.73	1.00	0.87	0.99
18.5 to <25.0	0.98	0.87	0.99	0.86	0.99
≥25.0	0.96	0.93	0.97	0.88	0.98
Smoking					
No	0.97	0.91	0.98	0.86	0.99
Current smoker	0.98	0.89	0.99	0.87	0.99
Heavy drinking^b^					
No	0.98	0.89	0.99	0.87	0.99
Heavy drinker	0.95	0.93	0.96	0.85	0.98
Blood pressure, mmHg					
Systolic < 120 and diastolic < 80	0.99	0.83	1.00	0.90	0.99
Systolic < 140 and/or diastolic < 90	0.97	0.93	0.98	0.88	0.99
Systolic < 160 and/or diastolic < 100	0.94	0.93	0.95	0.84	0.98
Systolic ≥ 160 or diastolic ≥ 100	0.92	0.84	0.94	0.80	0.95

Abbreviations: BMI, body mass index; NPV, negative predictive value; PPV, positive predictive value.

^a^ Prescriptions of antihypertensive drugs.

^b^ Heavy drinker defined as consumption of more than 46 g of ethanol per day.

**Table 5:** Validity of self‐reported use of medication for diabetes for predicting actual prescriptions over 3 mo among subgroups

	Diabetes^a^				
	Agreement	Sensitivity	Specificity	PPV	NPV
All	0.99	0.89	1.00	0.89	1.00
Sex					
Men	0.99	0.89	1.00	0.89	1.00
Women	1.00	0.87	1.00	0.92	1.00
Age, y					
<40	1.00	0.92	1.00	0.83	1.00
40 to 49	1.00	0.89	1.00	0.90	1.00
≥50	0.99	0.89	0.99	0.89	0.99
BMI, kg/m^2^					
<18.5	1.00	0.82	1.00	0.90	1.00
18.5 to <25.0	1.00	0.90	1.00	0.88	1.00
≥25.0	0.99	0.89	0.99	0.91	0.99
Smoking					
No	0.99	0.90	0.99	0.86	1.00
Current smoker	1.00	0.89	1.00	0.91	1.00
Heavy drinking^b^					
No	0.99	0.90	1.00	0.90	1.00
Heavy drinker	0.99	0.85	1.00	0.88	0.99
Hemoglobin A1c, %					
<6.5	1.00	0.84	1.00	0.88	1.00
6.5 to <8.0	0.91	0.92	0.89	0.91	0.91
≥8.0	0.87	0.92	0.80	0.87	0.87

Abbreviations: BMI, body mass index; NPV, negative predictive value; PPV, positive predictive value.

^a^ Prescriptions of oral hypoglycemic drugs and/or self‐injected insulin.

^b^ Heavy drinker defined as consumption of more than 46 g of ethanol per day.

**Table 6:** Validity of self‐reported use of medication for dyslipidemia for predicting actual prescriptions over 3 mo among subgroups

	Dyslipidemia^a^				
	Agreement	Sensitivity	Specificity	PPV	NPV
All	0.98	0.86	0.99	0.83	0.99
Sex					
Men	0.97	0.86	0.98	0.83	0.99
Women	0.99	0.87	1.00	0.85	1.00
Age, y					
<40	1.00	0.77	1.00	0.76	1.00
40 to 49	0.98	0.83	0.99	0.83	0.99
≥50	0.96	0.87	0.97	0.84	0.98
BMI, kg/m^2^					
<18.5	1.00	0.91	1.00	0.91	1.00
18.5 to <25.0	0.98	0.87	0.99	0.82	0.99
≥25.0	0.96	0.85	0.97	0.85	0.97
Smoking					
No	0.98	0.83	0.99	0.83	0.99
Current smoker	0.98	0.87	0.99	0.84	0.99
Heavy drinking^b^					
No	0.98	0.86	0.99	0.83	0.99
Heavy drinker	0.97	0.83	0.98	0.83	0.98
LDL cholesterol, mg/dL					
<120	0.98	0.87	0.99	0.86	0.99
120 to <140	0.98	0.88	0.99	0.85	0.99
140 to <160	0.98	0.83	0.99	0.79	0.99
≥160	0.97	0.75	0.98	0.62	0.99

Abbreviations: BMI, body mass index; LDL, low‐density lipoprotein; NPV, negative predictive value; PPV, positive predictive value.

^a^ Prescriptions of hypercholesterolemia drugs.

^b^ Heavy drinker defined as consumption of more than 46 g of ethanol per day.

## 4 DISCUSSION

In this study, we found that self‐reports of medication use among employees in large‐sized Japanese companies had high validity with actual prescriptions. In particular, agreement, sensitivity, specificity, PPV, NPV and kappa statistics showed markedly high validity against 3‐month claims data. To our knowledge, this is the first study to examine the validity of self‐reported medication use in an occupational setting in Japan.

To date, only one study has examined the validity of self‐reported medication use for hypertension, diabetes, and dyslipidemia in Japan. Fujita et al^1^ conducted a validity assessment of self‐reported medication use for hypertension, diabetes, and dyslipidemia in 54 712 participants aged 40 to 74 years who were beneficiaries of the National Health Insurance of Chiba City, Japan. The questionnaire phrasing (in Japanese) used in their study was exactly as the same as in ours, and was derived from a standard questionnaire initiated by the Ministry of Health, Labour, and Welfare, Japan, for specified health checkups initiated in April 2008.^17^, ^18^ This study also found high sensitivity and specificity scores between self‐reports and insurance claims covering 3 months, of namely 0.92 and 0.86 for hypertension, 0.83 and 0.99 for diabetes, and 0.86 and 0.91 for dyslipidemia, respectively, and thus quite similar to those in our study. The kappa values for hypertension, diabetes, and dyslipidemia medication use were 0.71, 0.77, and 0.70, whereas our data showed 0.87, 0.89, and 0.83, respectively. This discrepancy may due to the different prevalence of medication use due to the differences in the age structure of the two populations.^19^ There are also possible effects of difference in characteristics, such as socioeconomic factors (eg, education, occupation) and health status regarding transfer to regional health insurance due to retirement.^1^, ^17^ Our results support the high validity of self‐reported medication use in Japan, whether in regional or occupational populations.

We found the highest kappa values in the 3‐month reference period for claims data in hypertension, diabetes, and dyslipidemia. A similar finding was seen in the previous study in Chiba City.^1^ This may be due to the fact that physicians give relatively long‐term prescriptions for patients with stable chronic diseases. There is a report that the proportion of outpatients who had visited the hospital within 30 days of their last visit was 91.2% in 1996, but had fallen to 74.4% in 2014.^20^ Our present results showed sufficient validity for a reference period of 2 months or longer. Occupational health professionals accessing health insurance claims data should consider a 2‐ or 3‐month fixed look‐back period sufficient.^3^, ^21^ Given the reference period of one year, it is possible that the participants were intervened in by the results of the health checkup. This is reflected in the relatively high numbers of participants with no self‐report but with prescription in the 1‐year than other reference period results (Table 2).

Several studies have reported that discordance between self‐reported and gold standard medication use differed by individual level characteristics, such as age and sex.^11^, ^22^, ^23^ In the subgroup analyses of our study, we saw no major discordance, although sensitivity of medication use for hypertension was lowest among participants with younger age and leaner physique. Also, the PPV of medication use for dyslipidemia tend to be low among those with high LDL cholesterol. Further evaluation of this finding is challenging, however, given that a certain proportion of people reported negatively in self‐reports even though they actually had a prescription. We speculate on the presence of some unidentified bias, such as reporting bias.^9^, ^24^ We originally hypothesized that the degree of agreement would differ according to the severity of hypertension, diabetes, and dyslipidemia, but found only slight differences. Further studies are needed to detect differences in concordance by clinical or sociodemographic characteristics.

The strength of this study is its use of large cross‐sectional data, which are available through legally required health checkups for all employees in Japan.^18^, ^25^ Furthermore, combining these data with those from corporate health insurance unions, which typically enroll all the employees of a company, allowed us to verify the validity of the self‐reports. Additionally, since hypertension, diabetes, and dyslipidemia are routinely treated with drugs or self‐injected insulin prescribed by medical doctors, instead of with over‐the‐counter drugs,^26^ it was possible to obtain highly accurate information on actual medication rates.

Nevertheless, several limitations and bias might have affected our findings. First, our participants were employees of large‐sized companies, raising the issue of generalization depending on company size and type. However, our results were similar to those in local residents, supporting both the high validity and generalizability of our findings. Second, we analyzed only for hypertension, diabetes, and dyslipidemia, and medication for other diseases, such as mental disorders, respiratory diseases, and so on should also be considered. Third, we did not consider the patient compliance with medication—even if prescribed—medicine might still not be taken.^27^ Concordance of compliance measurement must also be examined.^28^, ^29^ Fourth, some doctors do not prescribe medicine for patients with hypertension, diabetes, or dyslipidemia even when these are diagnosed.^30^ We therefore conducted the same analysis for data on clinical diagnoses in place of prescriptions, but found no major differences (data not shown). Finally, information was limited to a single year. A longer follow‐up survey is now underway.

In conclusion, we found that validation of self‐reported medication use for hypertension, diabetes, and dyslipidemia was almost perfect among employees of large‐sized companies in Japan. The results of this study support the fact that occupational health professionals can rely on the results of self‐reported medication use for hypertension, diabetes, and dyslipidemia in carrying out health management and developing occupational health activities in companies.

## DISCLOSURE

Ethical approval: The study was approved by the ethics committee of the University of Occupational and Environmental Health, Kitakyushu, Japan (Protocol Number H26‐026).

Informed consent: Written informed consent was obtained from each participant.

Registry and the registration number of the study/trial: N/A.

Animal studies: N/A.

## CONFLICT OF INTEREST

The authors have no conflicts of interest directly relevant to the content of this article.

## AUTHOR CONTRIBUTIONS

KoF, TN, and KM conceived the study; TN, KM, and MN collected the data; KoF, with support from TN and MO, analyzed the data; and KoF led the writing. All authors participated in critically reviewing the paper.
